# From Cells to Organoids: Approaches, Regulatory Mechanisms, Applications, and Challenges of Organoids

**DOI:** 10.3390/cells14231898

**Published:** 2025-11-29

**Authors:** Lihong Wang, Anqi Chen, Dong Zhang, Zuping He

**Affiliations:** 1 Key Laboratory of Reproductive Health Diseases Research and Translation, Ministry of Education, NHC Key Laboratory of Tropical Disease Control, Hainan Academy of Medical Sciences, Hainan Medical University School of Life Sciences and Medical Technology, Haikou 571199, China; 2 Key Laboratory of Model Animals and Stem Cell Biology in Hunan Province, Engineering Research Center of Reproduction and Translational Medicine of Hunan Province, Hunan Normal University School of Basic Medicine, Changsha 410013, China

**Keywords:** organoids, molecular mechanisms, biomedicine, regenerative medicine

## Abstract

**Highlights:**

**What are the main findings?**

- This article systematically discusses the construction approaches, regulatory mechanisms, significant applications, perspectives, and challenges of organoids.

**What are the implications of the main findings?**

- It facilitates the development of organoids in unveiling organogenesis mechanisms and disease etiology.
- It promotes the application and development of organoids in drug screening and toxicity testing.
- It lays a foundation for the development of organoids in personalized medicine, regenerative medicine, and alternatives to animal experiments.

**Abstract:**

Organoids refer to three-dimensional (3D) multicellular tissues derived from stem cells or single cells through their self-assembly capacity, and significantly, they mimic structural and functional characteristics of the organ from which they are derived. Organoids can maintain the gene expression profiles and mutational features of parental cells during long-term culture. This makes organoids more relevant to the human bodies than gene knockout or overexpression animal models. Consequently, organoids have been widely used in various kinds of fields, including studies on organ developmental mechanisms, regenerative medicine, organ repair, the construction of disease models, high-throughput drug screening, and personalized medicine. Notably, significant progress has recently been made in organoid construction methodologies and regulatory mechanisms. These include the selections of starting cell sources, optimizing matrix materials, and the related cell signaling pathways. The rapid development of organoid technologies has provided new opportunities for their applications in organ transplantation, drug and toxicity screening, and molecular mechanisms for cell and tissue development. In this review, we discuss organoid construction methods involving the starting cell selection and spatiotemporal mediation, regulatory mechanisms with signaling molecules and pathways, and their applications in unveiling organogenesis mechanisms and disease etiology, drug screening, toxicity testing, personalized medicine, regenerative medicine, and alternatives to animal experiments. We also address the perspectives and challenges in this field with an aim to promote the development of organoids in basic research and translational medicine.

## 1. Introduction

Organoids, by definition, are three-dimensional (3D) structures with the capacity to mimic the structure and function of the organ from which they are derived [1]. They are generated by stem cells or other single cells under specific culture conditions. Compared with traditional two-dimensional (2D) cell culture, 3D cell culture can more authentically simulate the physiological environment of tissues in vivo. They are distinguished by three key features: controllable cell sources (e.g., well-characterized stem cells or patient-derived cells with a defined genetic background), standardized culture conditions, matrigel or other extracellular matrices, and precise and dynamic detection indicators that monitor cellular behaviors (e.g., proliferation and differentiation) or functional outputs (e.g., metabolite secretion) over time [2,3]. These characteristics enable them to reflect human organs more accurately and be more clinically relevant. Additionally, organoids’ reproducibility and high-throughput nature make them an ideal model for drug development and toxicological evaluation [4,5]. It is worth noting that organoids can be applied not only in basic research to uncover molecular mechanisms underlying organ physiology but also in personalized therapy in preclinical studies. For example, patient-derived organoids (PDOs) may be employed to evaluate how individuals respond to drugs, thereby assisting clinical decisions for specific patients [6,7].

**Figure 1:**
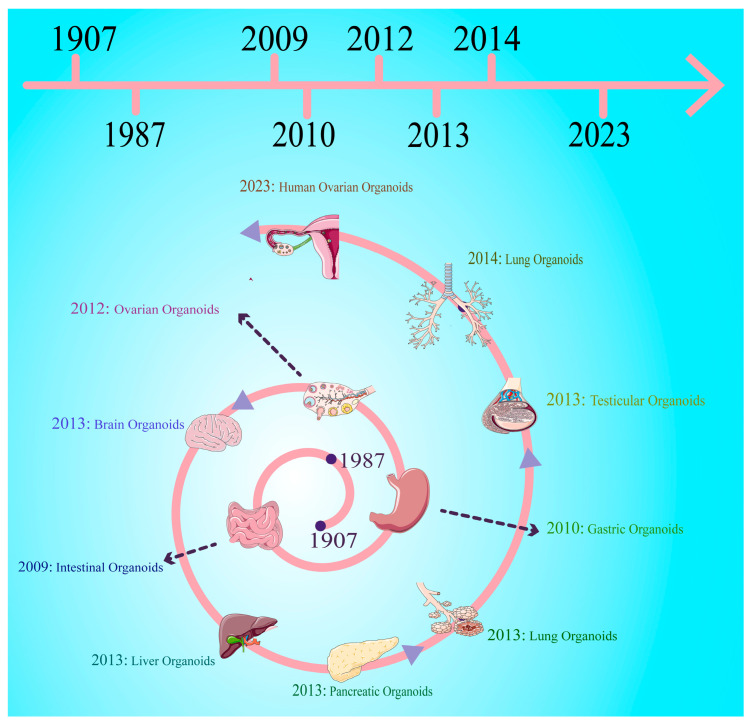
Timeline and advancement in organoid development and technologies. This diagram illustrates the milestones in the development of human organoids from 1907 to 2023 and highlights the progress made in constructing organoids that mimic the organ from which they are derived, including the intestine, brain, testis, liver, pancreatic, lung, and ovary. This progress has been achieved through stem cell research and tissue engineering.

The origin of cell culture technologies can be traced back to the late 19th century. Initial approaches involved 2D cell culture, which utilizes a nutrient medium as the energy source. While this method lays the groundwork for cell biology research, it is inadequate for simulating the in vivo environment of tissues. Progress has been made in simulating the physiological states and microenvironment of cells more effectively by transitioning to 3D cell culture techniques, e.g., suspension culture and matrix gel culture. This advancement has significantly fostered cell-cell interactions and signal transduction [8]. Mouse embryonic stem cells (mESCs) cultured in 3D systems are more likely to differentiate into cortical progenitor cells and functional projection neurons compared to those from 2D culture [9], and they can recapitulate the process of embryonic corticogenesis in vitro [9]. In 1907, it was reported that mechanically separated sponge cells can self-assemble to generate functional organoids. The organizational capacity of single cells is the crucial basis for the formation of organoids [10]. In the 1970s, it was found that co-culture of primary human keratinocytes and mouse fibroblasts can form a typically stratified squamous epithelial structure with the dividing cells in the basal layer and the differentiated keratinocytes in the apical layer, which lays the foundation for organoid research [11]. In 2009, Hans Clevers and colleagues isolated Lgr5^+^ stem cells from the bases of intestinal crypts and induced them to differentiate intointestinal organoids [1], which marks the beginning of studies on intestinal organoids. Since then, 3D cell culture technologies have enabled researchers to generate organoids from various tissue sources, including the liver, stomach, and testis [12,13,14], as we illustrate in Figure 1. These organoids structurally and functionally mimic the organ from which they are derived, thus demonstrating great potential in drug development, toxicity testing, and disease modeling. In this review, we focus on the construction methods of organoids and regulatory mechanisms that are involved in organoid formation. This study can clarify the biological relevance and clinical application values of organoids to offer new strategies for regenerative medicine via organ transplantation and uncovering pathogenesis of diseases.

## 2. Methods of Organoid Construction

Organoids are 3D-structured tissues formed by the aggregation of stem cells or other single cells via in vitro culture. The efficiency of organoid formation is closely related to the factors, including cell selection and cultivation, the microenvironment, cellular self-organization, and 3D structure. There are three main methods for organoid construction, namely, 3D cell culture, 3D bioprinting, and organoid-on-a-chip technology, and we discuss the differences between these methods, as summarized in Table 1.

### 2.1. Selection and Culture of Cells

The starting cells for organoid construction mainly originate from embryonic stem cells (ESCs), induced pluripotent stem cells (iPSCs), adult stem cells (ASCs), and patient-derived cells. The structural fidelity and functional maturation of organoids are determined by their multipotent differentiation capacity or tissue-specific differentiation potential [15,16]. Patient-derived organoids (PDOs) and patient-derived tumor organoids (PDTOs) typically involve patient-derived tissues with the histological and genetic characteristics of the primary tumor due to their inherent properties.

#### 2.1.1. Pluripotent Stem Cells (PSCs): ESCs and iPSCs

In 1981, mouse ESC lines were isolated from the inner cell mass (ICM) of blastocysts [22], and human ESC lines were established in 1998 [23]. ESCs possess the potential of an unlimited proliferation ability and differentiation into all cell types of body [24], which lays the foundation for the development of organoid technology. However, ethical concerns regarding embryo destruction and the risk of immune rejection limit their application in practice [25,26].

In 2006, iPSCs were generated from mouse fibroblasts by reprogramming via overexpressing four transcription factors, i.e., Oct3/4, Sox2, c-Myc, and Klf4 [27]. As an important cell source for producing organoids, iPSCs have several advantages. Firstly, iPSCs can be derived from autologous somatic cells of patients, which circumvents ethical issues and immune rejection risk. Secondly, iPSCs can express ESC markers and possess the potential to differentiate into cells of all three germ layers (endoderm, mesoderm, and ectoderm) [28,29,30], indicating that they can be used for obtaining numerous kinds of stem cells for generating organoids. Thirdly, iPSCs provide new ways for disease modeling and clinical trial when they are derived from specific patients. For instance, iPSC lines can be treated with inducible ETS translocation variant 2 (ETV2) and genetic modification via minicircle vectors [31,32]. On the other hand, iPSCs have issues similar to those of ESCs, e.g., tumorigenicity and epigenetic instability [33].

PSCs should be cultured in suspension conditions in order to form embryoid bodies (EBs), which mimic the cellular organization and intercellular signaling interactions of early embryos [34,35]. EB formation involves a transition from epithelial-like structures to 3D tissues, which is accompanied by the upregulation of specific gene expression. This lays the foundation for subsequent tissue- and organ-specific differentiation [36]. Gastric organoids require the differentiation of PSCs into neural ectoderm cells, mesenchymal cells, and epithelial progenitor cells (endoderm cells) to be assembled into functional structures [37,38]. Recent research shows that human gastrulating stem cells (hGaSCs), established from human pluripotent stem cells (hPSCs) as the starting cells, possess the ability to stably differentiate into multiple gastrulation-stage cell types. These stem cells can be stably passaged in vitro and differentiate into various gastrulation-stage cell types, such as endoderm-, mesoderm-, ectoderm-, amniotic ectoderm-, and primordial germ cell-like cells. These organoids provide a breakthrough in vitro model for studying early human embryonic development, disease mechanisms, and drug teratogenicity screening [39].

#### 2.1.2. Adult Stem Cells (ASCs)

ASCs are another cell source utilized for organoid generation. The construction process of organoids using ASCs differs from that using PSCs, and it uses tissue-resident stem cells with high self-renewal capacity and synergistic interactions with somatic cells to achieve organoid structures and functions [40]. ASCs are derived from mature tissues, e.g., intestinal crypts and the liver, via enzymatic digestion or mechanical isolation. They possess tissue-specific self-renewal and differentiation capabilities, which enables them to self-organize into organoids. The advantages of using ASCs for generating organoids include differentiation pathways closely resembling the organ from which they are derived, genetic stability, and the lower risk of immune rejection due to autologous sources [1,41], However, heterogeneity exists among ASCs from different tissue origins, which is influenced by donor age, sex, and tissue-specific factors [42].

#### 2.1.3. Patient-Derived Cells

Patient-derived tissues are obtained from patient samples (e.g., surgically resected or biopsy tissues). These tissues are processed into single-cell suspensions via enzymatic digestion or fluorescence-activated cell sorting (FACS), ensuring the preservation and purification of key cancer cells, including cancer stem cells (CSCs) [43,44]. The histological structure, genetic mutation profile, and drug sensitivity characteristics of cells in PDOs/PDTOs mirror those of the primary tumor. Moreover, CSCs can self-assemble into organoids when they are cultivated under specific in vitro conditions, making them feasible in creating precision tumor models [45].

### 2.2. The Effect of the Microenvironment or Niche on Organoid Development

The microenvironment serves as the core regulatory system for the in vitro development and functional maintenance of organoids. It integrates the physical signals and chemical messages sent out by organs to help organoids develop, grow, and work properly.

#### 2.2.1. Regulation of Physical Cues Stabilizes the Organoid Microenvironment

Physical cues provide cells with spatial layout, material exchange conditions, and signal regulatory environment via three ways: scaffold-free models, scaffold-based models, and mechanical force regulation. The core of scaffold-free models lies in cells that utilize their robust self-assembly capabilities to form functional structures of organoids without the need for exogenous scaffolds. For example, adipose-derived mesenchymal stem cells (MSCs) self-assemble to become cup-shaped organoids that exhibit more efficient diffusion of nutrients and oxygen to ensure the metabolism of internal cells [46]. As a promising strategy for repairing the damaged tissues and reconstructing organ functions, cartilage tissue engineering has attracted extensive attention in recent years. It mainly promotes the regeneration of damaged sites by combining living chondrocytes with degradable scaffold materials. However, traditional scaffold-based cartilage regeneration technologies have certain limitations since scaffold materials may trigger immune responses. A relevant study has proposed a novel scaffold-free three-dimensional cartilage regeneration technology based on cartilaginous organoid bioassembly (COBA) [47]. Primary chondrocytes are selected and they spontaneously aggregate to form spheroids [47]. After in vitro culture and expansion, these spheroids are cultured with growth factors until the cartilage tissues reach basic maturity [47]. This technology avoids potential immune responses caused by scaffold materials and the impact of degradation products on regeneration quality. Scaffold-based models offer mechanical support through exogenous scaffolds that enable organoids to maintain physiologically spherical morphology and promote cell differentiation and organoid maturation. For instance, kidney decellularized extracellular matrix (dECM) hydrogel scaffolds can provide physical support and mimic the in vivo microenvironment through ECM components, which enhances the filtration function of kidney organoids [48]. Matrigel has been employed by us as a core scaffold material to provide physical support and a physiological microenvironment, which enables male germ cells and somatic cells to establish testicular organoids that simulate the process of spermatogenesis in vivo [49]. This refines the regulatory networks of the testicular microenvironment which is essential for testis organoids.

#### 2.2.2. Signaling Pathways in Regulating Fate Specification of Organoids

The assembly and maturation of organoids are accomplished through the regulation of signaling molecules. In August 2021, human cardiac organoids were generated through self-assembly using human PSCs [50]. The Wnt pathway agonist CHIR99021 activates the canonical WNT/catenin pathway and initiates the differentiation of mesoderm into the cardiac lineage [50]. Meanwhile, the complexity of human cardiac organoids (hHOs) increases due to the induction of pro-epicardial organ formation. The addition of BMP4 and Activin A improves the cavity formation and vascularization of cardiac organoids [50]. By spatiotemporally specific activation or inhibition of specific signaling molecules and pathways, precise regulation of cell fate determinations of organoids can be achieved from multiple dimensions.

### 2.3. Self-Assembly and 3D Structure Formation of Organoids

The 3D structures of organoids can be formed by stem cells, which involves signals inside the cells, cell-cell interactions, matrix interactions, cell proliferation and migration, and the perception of mechanical forces. It has been demonstrated that the physical properties of the extracellular matrix, e.g., stiffness and adhesiveness, the impact of cell self-assembly, and modulating the mechanical properties of the matrix, can effectively control the morphology and function of organoids [51]. Moreover, the self-assembly process of cells is regulated by growth factors and cytokines that promote their differentiation and organization by activating specific signaling pathways [17]. Human urine-derived stem cells (USCs) are seeded onto a porous 3D silk fibroin scaffold and cultured in an osteogenic differentiation medium to induce cell self-assembly to form bone tissues [52]. The 3D culture system for organoids primarily relies on biological scaffolds, including matrigel and collagen, to simulate the physicochemical properties of the in vivo ECM and provide necessary mechanical support [16]. The 3D culture systems can be classified into two main forms, including static culture and dynamic perfusion (e.g., microfluidic chips) [53,54]. Liver organoids, for instance, generated by the micropillar chip, display more comprehensive liver function characteristics [55]. Moreover, cell-based 3D and tissue slice-based 3D co-culture might be used for organoid generation [56].

Organoid culture relies heavily on matrigel. Matrix components extracted from mouse tumors can mimic the stem cell niche, and thus they have significant limitations since their chemical composition is undefined. The single-chain activating antibody TS2/16 (scTS2/16) retains the variable regions that recognize and activate integrin β1. The variable domains of the heavy and light chains are connected by a flexible linker peptide, and it has been reported to efficiently activate integrin β1 on the cell surface, which enhances the growth of organoids in both matrigel and collagen hydrogels [57]. This research paves the way for the standardized and large-scale culture of organoids, and ultimately, their application marks the entry of organoid culture into a new era of defined composition and clinical applicability [57].

### 2.4. Maturation Identification and Functional Characterization of Organoids

The maturation of organoids usually takes several weeks or months. Factors, including cell source, culture conditions, and operational techniques, affect generation efficiency (ranging from 30% to 80%) and maturation of organoids [58]. Identification of mature organoids requires an analysis from multiple dimensions. Histological analysis verifies structural similarity to the organ from which they are derived [17]. Functional assessment detects specific indicators for functions, e.g., albumin secretion and drug metabolism capacity for liver organoids [16]. Molecular characterization compares organoids’ consistency with that of the organ from which they are generated through transcriptomics and proteomics [59], while safety assessment focuses on genetic stability and tumorigenicity risk [60].

The values of organoids depend on their physiological functions, including cell proliferation, differentiation, metabolic activity, and response to external stimuli [61]. Liver organoids can assume energy metabolism, drug metabolism, and toxicity reactions by their hepatocytes [62], and they might be applied to unveil the pathogenesis underlying liver inflammation and hepatocellular carcinoma to identify therapeutic targets for these diseases [63]. Currently, the main challenges in functional characterization of organoids lie in standardizing organoid culture systems and assessment methods to ensure reproducibility and reliability across different laboratories [64].

### 2.5. Organoid Types

Organoids can be categorized into multiple types based on their sources, including adult stem cell-derived organoids (ASCOs) originated from adult tissues [40], pluripotent stem cell-derived organoids (PSCOs) derived from embryos [24] or pluripotent stem cells [27], PDTOs directly obtained from patient tissues [44], assembloids constructed by combining multiple organoids [65,66], gastruloids with focus on simulating early-stage development [39], and organoid-on-a-chip system engineering platforms integrated with organoids [67,68,69]. Different types of organoids have distinct applications, e.g., personalized medicine for ASCOs and PDTOs [1,41,45], research on developmental mechanisms and diseases for PSCOs [70], simulating complex cell-cell interactions for assembloids [65,66], studies on early-stage vascularization for gastruloids [71], and dynamic control and drug testing for organoid-on-a-chip systems [72]. In terms of core technologies, organoid-on-a-chip systems rely on microfluidic methods to achieve precise control. Other types of organoids place emphasis on the self-organization ability and differentiation potential of cells. Regarding integration potential, organoid-on-a-chip systems can be adapted to multiple types of organoids (e.g., PDTOs, gastrula organoids). Assembloids can also serve as core input units. We compare different types of organoids in Table 2.

## 3. Regulatory Mechanisms Underlying Organoid Development

**Figure 2:**
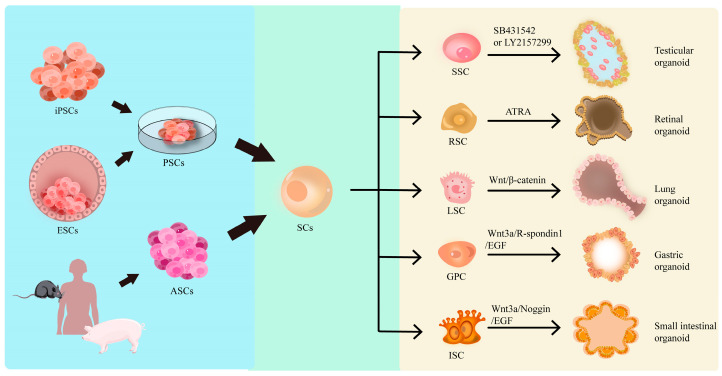
Signaling factors and pathways for organoid development. This diagram demonstrates how various kinds of organoids can be generated from different stem cells by using specific factors. ESCs: embryonic stem cells; iPSCs: induced pluripotent stem cells; PSCs: pluripotent stem cells; ASCs: adult stem cells; SCs: stem cells; SSCs: spermatogonial stem cells; RSCs: retinal stem cells; LSCs: lung stem cells; GPCs: gastric progenitor cells; ISCs: intestinal stem cells.

The regulatory mechanisms of organoid development constitute a multidimensional and dynamically coordinated system that can be categorized into four modulations, including cell-autonomous regulation, extrinsic microenvironmental regulation, spatiotemporal dynamic regulation, and metabolic/mechanical force regulation, as we discuss and illustrate in Figure 2.

### 3.1. Cell-Autonomous Regulation

The process of cell-autonomous regulation depends on the genome’s inherently developmental programs. These programs dictate the direction of cellular differentiation through the mechanisms of transcriptional regulation and epigenetic modifications, which lays the basis of organoid formation. The differentiation of distinct organoids relies on specialized transcription factors whose functions exhibit strict tissue specificity and stage dependency. CRISPR screening of adult intestinal organoids indicates that transcription factor ZNF800 specifically suppresses endocrine progenitor cell differentiation into enterochromaffin cells [78]. In gastric organoids, MYC stabilizes EPCAM to induce metabolic reprogramming, which inhibits lysosomal biogenesis through the activation of mTOR and ERK [79]. This process prevents the degradation of EPCAM via the macropinocytosis pathway, thereby promoting the chromatin accumulation of β-Catenin and the activation of WNT oncogenic transcription [79]. Testicular organoids involve the interaction of male germ cells and somatic cells to form regulatory networks. The maintenance of SSCs relies on PLZF to sustain their self-renewal [14]. In human-derived organoids and metastatic colorectal cancer (CRC), the squamous-like expression signature induced by Atrx deletion is closely associated with reduced expression of lineage factors, chromatin remodeling, increased invasiveness, and poor prognosis [80]. Sumoylated SnoN acts via the regulation of histone deacetylation HDAC1 to modulate EMT-related effect in breast organoids [81]. The overexpression of the miR-17-92 cluster is associated with malignant progression in colorectal adenoma organoids [82].

### 3.2. Regulation of the Extracellular Microenvironment (ECM) in Organoids

The ECM regulates organoid morphogenesis by providing physical support and anchoring signals. The organoid-specific composition and stiffness of the ECM directly determine their morphological differentiation. For instance, intestine organoids depend on matrigel alone, while gastric organoids require matrigel and collagen I. Lung organoids rely on matrigel and fibrin. By mimicking the physical properties of the in vivo organ microenvironment, these ECM components fulfill core functions, including the maintenance of epithelial polarity, promotion of parietal cell polarization, and support of airway branching [83,84,85]. The maturation and functional performance of hepatic organoids depend heavily on the stiffness characteristics of the ECM. It has been shown that choosing an ECM with a stiffness of 1–10 kPa (e.g., matrigel or certain synthetic hydrogels) is essential for facilitating hepatocyte interactions and detoxification functions, which is crucial for developing functional liver organoids [86]. To replicate the core functions of testicular organoids in mimicking the testis in vivo, it is necessary to composite ECM scaffolds with matrigel and collagen IV [87], which offers essentially physical support and signal anchoring for testis organoids to maintain seminiferous tubule-like structures and promote the directional differentiation of male germ cells [87].

### 3.3. Tissue-Specific Signaling Pathways and Intercellular Interactions

The directed induction of organoids lies in the specificity of the “activation or inhibition combination” of classical signaling pathways that are involved in in vivo organogenesis. For intestinal organoids, Wnt3a/Noggin/EGF maintains the Lgr5^+^ stem cell pool and inhibits differentiation to form crypt-villus structures [1]. In the development of testicular organoids, small-molecule inhibitors, e.g., SB431542 and LY2157299, have been shown to promote spermatogonial proliferation and optimize cell-cell interactions and structural reorganization by inactivating the TGF-β signaling pathway [87].

Organoid maturation relies on intercellular crosstalk. In vitro vascularized organoids have been generated to reproduce the synergistic development of mesodermal and endodermal lineages, and cells within organoids spontaneously secrete signaling molecules essential for vascular growth to form vascular networks [88]. This enables the lung and intestinal organoids to develop organ-specific endothelial and mesenchymal components, which enhances cellular diversity, 3D architecture, cell viability, and maturation with physiological functions [88].

### 3.4. Spatiotemporal Regulation of Organoids

Organoids develop through distinct developmental stages (induction, proliferation, differentiation, and maturation) and switch stage-specific signals in the temporal dimension. This stage-specific dependency on regulatory cues is particularly evident in retinal and corneal organoids. In retinal/corneal organoids, stage-specific functions are exhibited by all-trans retinoic acid (ATRA) [89]. Corneal organoid transparency, a hallmark of the induction stage, is promoted by high concentrations of ATRA, whereas photoreceptor maturation is persistently suppressed, which causes defect in the maturation stage [89]. Conversely, low concentrations of ATRA enhance pigment deposition in the retinal pigment epithelium (RPE) and stimulate photoreceptor maturation [89]. Thyroid hormone T3 and taurine promote rod photoreceptor and red/green cone maturation [89], which indicates that each stage necessitates distinct combinations of signaling molecules.

Organoids also have the spatial zonation characteristics of the organ from which they are derived through differences in the levels of signaling molecules or gene expression. The crypt region of the small intestine has high levels of the Wnt protein [90], which is important for maintaining stem cells. In contrast, BMP is expressed highly in the villus region [90], which promotes epithelial differentiation. Together, Wnt and BMP proteins form a radial gradient that mediates cellular proliferation in the crypt and differentiation in the villus [90]. In the lung, the presence of FGF10 in the airway region causes bronchial branching [91,92]. Conversely, BMP4 in the alveolar region of the lung promotes alveolar epithelial differentiation [91,92], which establishes the “airway–alveolar” spatial patterning [91,92]. In gastric-associated structures, the superficial layer exhibits high expression of SOX2 [93,94], which sustains gastric epithelial identity. The deep layer displays high levels of SOX9 expression [93,94], which maintains gastric glandular progenitor cells. Together, they form a hierarchical “gastric lumen-to-gland” spatial architecture through differential gene or protein expression levels.

## 4. Applications of the Organoids Across the Spectrum of Biological Research

**Figure 3:**
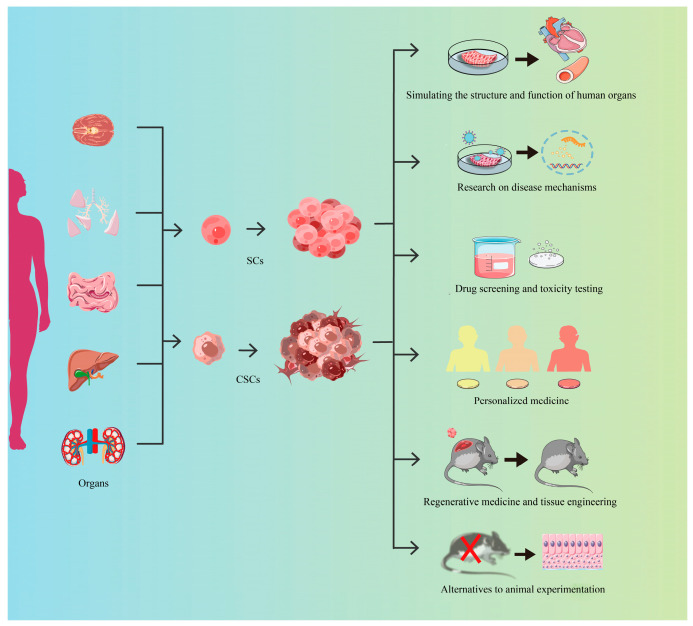
The applications of organoids in various kinds of fields. This diagram illustrates the utilization of cells derived from human organoids in multiple biomedical fields. SCs: stem cells, CSCs: cancer stem cells. “×”indicates Novel Alternative Methods (NAMs) utilizing organoids, which aim to replace and reduce animal experiments, along with their supporting implementation checklist.

Organoids possess tissue and organ structures and functions, owing to their high fidelity in mimicking organs from which they are derived, and thus they have demonstrated tremendous application potentials in life sciences and health, as we illustrate in Figure 3.

### 4.1. Simulating the Structures and Functions of Human Organs by Organoids

As the 3D in vitro models, organoids have unparalleled advantages over traditional cell and animal models for unveiling the mechanisms of organogenesis. Cerebral organoids are excellent models for investigating human brain development and brain-related disorders [95], while kidney organoids can be used to explore molecular mechanisms underlying renal development and genetic diseases [48]. Therefore, organoid models retain the functional characteristics of organs from which they are derived to provide unique platforms for the studies on organ morphogenesis and functional establishment.

### 4.2. Disease Mechanism Research Using Organoids

Organoids mimic the pathological processes of genetic, degenerative, infectious, and other diseases, enabling the exploration of disease etiology. ASCOs are co-cultured with vascular organoids, and these liver organoids further develop into vascularized liver microtissues. These microtissues successfully simulate the complex vascular network structure of the liver, thereby serving as a valuable platform for studying vascular abnormal pathology associated with liver diseases, such as liver cirrhosis and liver cancer [96]. In genetic diseases, organoids generated from patient-derived iPSCs can replicate disease phenotypes. Lung organoids derived from cystic fibrosis patients exhibit characteristic swelling due to dysfunction in chloride transport caused by *CFTR* mutation [97]. These organoids provide an excellent model for studying epithelial cell functional abnormalities and compensatory mechanisms [97]. By differentiating hPSCs into striatal organoids and midbrain organoids and assembling them into a basal ganglia circuit model (human Striatal-Midbrain Assembloids, hSMAs), the pathological propagation process of α-synuclein (α-syn) was successfully simulated. This model not only recapitulated the functions of the nigrostriatal and striatonigral pathways but also revealed the propagation mechanism of abnormal protein aggregation between neurons [98]. The heart-on-a-chip (HoC) platform integrates engineered 3D cardiac tissues with microfluidic technology to simulate the structures and function of cardiac tissues, thereby providing a new tool for studying the pathogenesis of cardiovascular diseases [99]. It has been demonstrated that patient-derived colonic organoid systems can be employed to unveil the mechanisms by which colonic-resident CD8^+^ T cells induce epithelial apoptosis and gut barrier disruption due to metabolic dysregulation in HIV-infected individuals receiving antiretroviral therapy (ART). Furthermore, activating the lipid metabolism factor PPARγ may restore immune cell metabolism and reduce epithelial damage [100], which offers new approaches for future clinical interventions of infectious diseases.

### 4.3. Drug Screening and Toxicity Testing Using Organoids

Brain organoids derived from iPSCs have become ideal models for neurodisease drug screening due to their high degree of similarity to the human brain. Organoids modeling Alzheimer’s disease (AD) are used to test the effect of drugs on neuronal function [101]. PDOs/PDTOs preserve tumor heterogeneity and genomic characteristics, making them suitable for anti-tumor drug screening. For example, melanoma organoids are used to monitor the efficacy of cisplatin and temozolomide via a 3D imaging system [102]. Assembloids simulate inter-organ interactions by combining different cell types. For instance, tumor-neural assembloids are applied to study the mechanisms of cancer neural invasion and the influence of drug intervention [103]. The microfluidic technology in organoid-on-chip systems enhances the physiological relevance of organoids. In personalized drug screening for breast cancer and liver cancer patients, the chip enables simultaneous dual evaluation of drug efficacy and toxicity, which is consistent with clinical pathology and ex vivo experiments [104].

### 4.4. Personalized Medicine Facilitated by the Use of Organoids

Patient-derived brain organoids can retain individual genetic variations, providing a platform for predicting personalized drug response [101]. Patient-derived liver organoids (e.g., pluripotent stem cell-derived liver organoids, PSC-LOs) support drug screening in precision medicine by simulating the authentic structure of the liver. Although current models have limitations in capturing epithelial heterogeneity, technological advancements are expected to enhance their clinical application potential [105,106]. The human ascending somatosensory assembloid (hASA) model, derived from patient iPSCs, can simulate individual-specific pathological features. It provides a tool for predicting personalized drug efficacy and evaluating toxicity, thereby advancing the application of precision medicine in the field of neurosensory diseases [66]. Patient-specific vascular-on-a-chip systems are used to study individual thrombus mechanisms and develop vascular devices [107].

### 4.5. Regenerative Medicine and Tissue Engineering of Organoids

Organoids have the potential to address the shortage of organ donors for transplantion, and they can also provide important cells or replacement tissues for organ repair. By orthogonally activating the transcription factors ETV2 and NKX3.1, researchers have established a simplified method for generating vascular organoids (VOs) from iPSCs, and VOs can be used to examine vascular development and the etiology of diseases; however, the independent regulation of endothelial cells and mural cells still needs optimization [108]. It enables the large-scale production and further maturation of organoids using bioreactors to optimize culture conditions. For instance, kidney organoids cultivated in dynamic bioreactors can form more complete glomerular and tubular structures, which exhibit the significantly enhanced renal function activities [109]. Combining organoids with biodegradable scaffolds can generate larger-scale functional tissues or organoids. For example, seeding intestinal organoids onto tubular biodegradable scaffolds can produce “mini-guts” with intact epithelial barrier function and peristaltic capability [110], which reflects potential for intestinal repair in patients with short bowel syndrome. Pancreatic organoids have the ability to differentiate into insulin-secreting islet β cells [111]. When transplanted into diabetic model mice, they effectively decrease blood glucose levels and regulate insulin secretion in response to changes in glucose levels [111], and this technology is currently at the preclinical research stage [111]. Retinal organoids can differentiate into high-purity retinal pigment epithelium (RPE) cells. The survival of photoreceptor cells can be maintained, and visual function can be improved by replacing damaged RPE cells with these cells after they are transplanted into the subretinal space of patients with age-related macular degeneration [112]. Clinical trials have demonstrated that HLA-matched allogeneic iPSC-RPE cell transplantation is safe and exhibits stable survival [113].

### 4.6. Novel Alternative Methods (NAMs) by the Use of Organoids

On 8 October 2025, the Center for Drug Evaluation and Research (CDER) of the U.S. Food and Drug Administration (FDA) officially released a position paper titled “Replacing and Reducing Animal Testing” and its supporting implementation checklist. This document not only provides actionable alternatives to animal testing for the biopharmaceutical industry but also marks a fundamental shift in the concept of nonclinical drug evaluation, which moves away from long-term reliance on animal models toward a new assessment system centered on human biological data. It thereby opens up broad application prospects for NAM technologies represented by organoids.

A new perspective review published by the European Federation of Pharmaceutical Industries and Associations (EFPIA) in Nature Reviews Drug Discovery puts forward targeted NAMs solutions. To address the issue of lack of target molecules in animals, a co-culture model of “tumor cells + T cells” can be constructed in vitro to directly simulate the activating effect of drugs on the immune system. By measuring indicators, such as cell killing activity and cytokine release, the safe starting dose for humans can be accurately calculated. To tackle the problem of interspecies differences in target molecules, human-derived and animal-derived vascular cell models can be compared to verify whether drugs cause specific damage to human vascular endothelium. Combined with computational models to predict the safety window, unnecessary animal tests can be reduced. For the challenge where the target is a non-mammalian protein, sequence alignment is used to ensure that the drug acts only on pathogen targets, thereby avoiding off-target effect in humans. Meanwhile, human hepatocyte models are utilized to verify metabolic safety and reduce the risk of liver injury. To deal with unexpected clinical toxicity, 3D liver organoid chips are employed to simulate bile acid metabolism, which reveals the mechanism by which drugs inhibit bile salt export pump (BSEP). Quantitative Systems Toxicology (QST) models are used to predict clinical risk and guide subsequent drug optimization [114].

### 4.7. The Challenges and Prospects of Translational Medicine with Organoids

Although organoids show great promise in drug screening and personalized medicine, their clinical applications still face certain challenges. Firstly, the creation and maintenance of organoids requires advanced techniques and equipment, which limits their widespread use to some extent [115]. Traditional organoid culture relies on an animal-derived matrix, such as matrigel, which has complex composition, large batch variation, and high price. Studies have shown that inorganic biomaterials, e.g., CS/GelMA composite hydrogels, have the advantages of biological activity, biosafety, and cost-effectiveness, and thus they can be used as high-quality substrates for organoid culture. These materials not only support the formation and development of organoids but also significantly reduce the cost of traditional medium and scaffolds [116]. In addition, the biomaterial driven vascularized culture system decreases the dependence of organoids on expensive growth factors by mimicking the in vivo microenvironment [95]. In traditional matrigel, the addition of scTS2/16 can increase the yield of all gastrointestinal organoids by up to 5 folds. More importantly, it significantly improves the clonogenic efficiency of single-cell seeding, which addresses the technical bottleneck in organoid establishment. In the well-defined type I collagen hydrogel, the effect of scTS2/16 is even more remarkable, which enhances the organoid yield by 6–7 folds [57]. Secondly, the biological differences between organoids and clinical samples may affect the predictive accuracy of drug response, highlighting the urgent need to improve their similarity to real tumor tissues [45]. Thirdly, ethical concerns and regulatory policies may hinder the clinical application of organoids [117]. Therefore, future research should focus on optimizing organoid culture techniques, standardizing operational procedures, and addressing ethical and legal issues, in order to facilitate the clinical applications of organoids [118]. Overcoming these challenges is expected to enable organoids to play greater roles in translational medicine, which will ultimately improve patient outcomes and quality of life.

## 5. Perspectives and Challenges of Organoids

### 5.1. Technological Advancement and the Use of Innovative Materials

The future development of organoid technology depends on technological advancement and the use of new materials, especially innovations in biomaterials and microfluidic technologies. In recent years, researchers have started to explore new biomaterials, e.g., calcium silicate nanowires and GelMA-based composite hydrogels modified with gelatin, which provide excellent substrates for organoid formation and functions. These new materials enhance the growth and survival rates of organoids and offer more biologically realistic environments for drug screening and disease modeling [116]. The production process of organoids has become more efficient and standardized by means of the application of microfluidic technology. This has paved the way for large-scale production, which in turn has advanced the development of personalized medicine [119]. With the development of novel technologies, organoids have emerged as powerful tools for unveiling the pathogenesis of diseases and developing new drugs [18].

### 5.2. Core Challenges and Breakthroughs in the Use of Organoids

The current core challenges of organoid technology mainly focus on key aspects, including vascularization, immune integration, functional maturation, and standardization.

#### 5.2.1. Core Technical Challenges

In terms of vascularization, bioengineering strategies, e.g., combinations of vascular inductive factors (e.g., VEGF, FGF) and microfluidic perfusion systems, have been developed. Nevertheless, it remains difficult to construct vascular networks with physiological-level branched structures and perfusion functions [120]. This directly leads to hypoxia and accumulation of metabolic wastes inside organoids, which limits their size expansion (usually <500 μm) and long-term culture stability [121,122]. Recent studies have attempted to activate the FAK/cofilin signaling pathway to promote vascular maturation through mechanical stimulation (e.g., fluid shear stress) or to monitor the vascularization process using biosensor chips. However, the spatial tissue precision is still insufficient [69,123].

Immune system integration faces dual challenges. On the one hand, the lack of a natural immune microenvironment (e.g., M cell–dendritic cell interaction in intestinal organoids) limits research on infection and immunity [124]. On the other hand, the issue of immune rejection after transplantation remains unresolved, which requires optimization through HLA matching or immune-privileged materials [125].

Standardization and scaling-up are key obstacles to the practical application of organoids. Although numerous studies have been dedicated to optimizing organoid culture conditions and methods, the lack of unified standards and operating procedures still limits their widespread application in clinical practice and drug development [115]. To address this concern, researchers are exploring the establishment of standardized production processes, including the unified protocols for material selection, culture medium composition, incubation time, and culture conditions. Standardizing the production process of organoids can enhance their reproducibility and reliability, making their applications more feasible in personalized and regenerative medicine [126].

#### 5.2.2. Technical Breakthroughs and Optimization Directions

The breakthrough of technical bottlenecks relies on interdisciplinary integration. Bioprinting can construct bionic vascular topological structures, but it is necessary to balance printing resolution and cell viability [127,128]. Organ-on-a-chip technology promotes the coordinated maturation of vascular-parenchymal cells through a mechanical-biochemical coupled microenvironment [69]. Meanwhile, it is necessary to define core evaluation indicators, such as vascular density and barrier function [120,129]. Optimization technologies can promote the application transformation of organoids in personalized medicine [130], regenerative medicine, drug screening, and disease modeling [131,132].

### 5.3. Ethical Issues and the Establishment of Regulatory Frameworks for Organoid Use

The rapid development of organoid technology has created an urgent need to address the associated ethical issues and establish regulatory frameworks. Organoid research involves the use of human cells and tissues, which raises concerns about ethical and legal issues (e.g., ensuring the informed consent of research subjects) and protects personal privacy [133]. While some countries and regions have formulated relevant ethical guidelines, there is currently a lack of unified regulatory frameworks and standards globally, which creates uncertainty around the conduct of organoid research [134]. Therefore, establishing a comprehensive ethical and regulatory framework would not only promote the healthy development of organoid technology but also provide the legal safeguards and ethical support for scientific research [135]. Such a framework should cover all aspects of research design, implementation, and reporting to ensure transparency and fairness, thereby enhancing public trust in organoid research [136].

## 6. Conclusions

Organoids are 3D multicellular constructs that can more authentically simulate the physiological environment of tissues in vivo, and they retain parental cell genetic and mutational features with superior relevance to human biology compared to traditional animal models. Advancements in starting cell selection, matrix optimization, and signaling pathway regulation have enhanced organoid construction and expanded their applications in organ development, disease modeling, drug screening, personalized medicine, and regenerative medicine (Figure S1). Current challenges include standardized culture protocols, insufficient organoid maturity, and scalability limitation, which need to be addressed through interdisciplinary collaboration. Future development of organoid technologies will further promote their translation from basic research to clinical practice, which drives progress in biomedical science and translational medicine.

## Figures and Tables

**Table 1:** Methods for organoid construction.

Culturing Technologies	Cell Types	Principles	Benefits	Application Fields	References
3D cell culture	ASCs/iPSCs	ASCs self-organize; iPSCs differentiate	High physiological relevance; personalized construction	The construction of liver, kidney, and intestinal organoid models; personalized medicine	[15,16]
		ECM scaffold materials like matrigel simulate the in vivo microenvironment and promote 3D cell self-organization	Provides a near-in vivo extracellular matrix environment and regulates stiffness and biocompatibility	Facilitation of the construction of intestinal and liver organoids and support for cancer research	[17]
		Self-formation of organoids	Replicates the natural development process	Developmental biology	[18]
		Multi-cellular co-culture for simulating intercellular interactions	Enhances organoid function	Immune responses, regeneration, viral infections, and other aspects of organs, e.g., the liver and kidneys	[19]
3D bioprinting		Printing layer by layer using bioink	Precisely controls the spatial distribution of cells to construct organ microstructures containing microvessels	Drug screening and regenerative medicine for liver organoids	[20]
Organoid-on-a- chip		A microfluidic organoid chip and microchannels mimicking vascular networks to offer a dynamic microenvironment for enhanced organoid functionality	Culture conditions can be dynamically controlled	Research on drug toxicity and immune regulation; construction of liver and lung organoids	[21]

**Table 2:** Core technologies and integration potential of different organoid types.

Organoid Types	Cell Sources	Supporting Matrix	Strengths	Limitations	Major Applications	References
ASCOs	ASCs	Matrigel/ECM/synthetic hydrogel	Rapidly established, preserving specific structure/function	Lineage differentiation potential is limited	Adult disease modeling, regenerative medicine	[73]
PSCOs	ESCs/iPSCs	Matrigel/ECM/synthetic hydrogel	Pluripotent differentiation, simulate early organ development	Long culture cycle, insufficient maturity	Developmental biology, genetic disease mechanisms, gene editing	[73,74,75]
PDOs/PDTOs	Patient- derived tissues	Matrigel	Preserve tumor genetics/microenvironment, high clinical relevance	Lack complete immune microenvironment (insufficient stroma cells)	Personalized tumor therapy, high-throughput drug screening	[44,45,76,77]
Assembloids	iPSCs	Matrigel/ECM/synthetic hydrogel	Simulate multi-tissue interactions, cross-tissue signaling	Complex technology, hard to standardize, coordinating developmental rhythm required	Research on tissue interactions, cross-organ signal transduction	[65,66]
Organoid-on-a- chip	Cells/tissues	Matrigel/synthetic hydrogel scaffold/chip	Dynamic physicochemical regulation, enhances vascularization/metabolism	High equipment dependence	Drug toxicity testing, permeability prediction, physiological simulation	[68,69,72]
Gastruloids	PSCs	Synthetic hydrogel scaffold/biomaterial	Simulate process of gastrulation in early embryos	Incomplete structure and function	Early development simulation, vascularization research	[39,71]

## Data Availability

Not applicable.

## Supplementary Materials

The following supporting information can be downloaded at: https://www.mdpi.com/article/10.3390/cells14231898/s1, Figure S1: This diagram shows the cell source, 3D culture, regulatory pathways, and applications of organoids.
