# Supplementary material for: From Cells to Organoids: Approaches, Regulatory Mechanisms, Applications, and Challenges of Organoids
Source: Cells. 2025 Nov 29;14(23):1898. doi: 10.3390/cells14231898 (PMC12691017; doi:10.3390/cells14231898)
Supplement: Supplementary file 1 [file cells-14-01898-s001.zip › cells-3971758-supplementary.pdf]

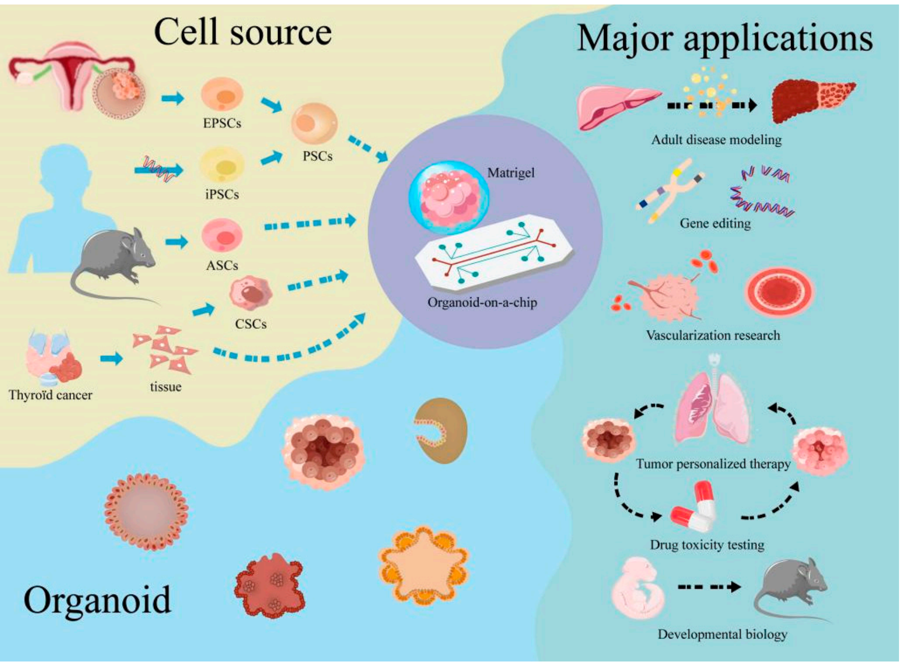

Figure S1. This diagram shows the cell source, 3D culture, regulatory pathways, and applications of organoids.
